# Advancing Refugee Health Data Management: The Implementation of ICD-11 in UNRWA’s Primary Care System

**DOI:** 10.3390/ijerph21091121

**Published:** 2024-08-26

**Authors:** Cassandra Broadwin, Wafa’a Zeidan, Mai Siam, Nenad Kostanjsek, Henry Victor Doctor, Eman Abdelkreem Aly, Mohammad Shraim, Ghada Ballout, Akhiro Seita

**Affiliations:** 1 United Nations Relief and Works Agency for Palestine Refugees, Amman 11814, Jordan; w.zedan@unrwa.org (W.Z.); m.siam2@unrwa.org (M.S.); m.shraim@unrwa.org (M.S.); g.ballout@unrwa.org (G.B.); a.seita@unrwa.org (A.S.); 2 World Health Organization, 1211 Geneva, Switzerland; kostanjsekn@who.int; 3 World Health Organization, Regional Office for the Eastern Mediterranean, Cairo 11371, Egypt; doctorh@who.int (H.V.D.); alye@who.int (E.A.A.)

**Keywords:** ICD-11, Jordan, Syria, Lebanon, Gaza, West Bank, refugee health, complex humanitarian setting, implementation science, global health informatics, health equity, gender minority health

## Abstract

The United Nations Relief and Works Agency for Palestine Refugees in the Near East (UNRWA) was one of the earliest healthcare systems globally to implement the International Classification of Diseases, Eleventh Revision (ICD-11) across its 140 clinics serving 5.9 million Palestine refugees. This paper discusses the integration of ICD-11 into UNRWA’s cloud-based electronic medical record (EMR) system, identifying both the barriers and facilitators involved and analyzing trends in clinical documentation and healthcare utilization. The key challenges included data privacy provisions, integration into a coordinated care model, complex classification schema for primary care settings, frequent staff turnover, and limited data analysis capabilities. Conversely, facilitators included physician-tailored training and on-site support, system compatibility, a multidisciplinary team approach, policy support from UNRWA and the World Health Organization (WHO), and leadership commitment and effective change management. Medical officers (MOs) using ICD-11 reported greater satisfaction with the system’s capabilities in managing and visualizing health information. This article contributes to the discourse on health data management in complex humanitarian settings, offering insights into the benefits and challenges of implementing advanced classification systems like ICD-11. Future research should explore longitudinal impacts and further integration with global health systems, ensuring that the advancements in classification continue to support the overarching goal of health equity and access in vulnerable and hard-to-reach populations.

## 1. Introduction

The integration of the International Classification of Diseases, Eleventh Revision (ICD-11) into health systems represents a significant step forward in the standardization of health data classification. Originating in the 19th century, the latest version of the ICD, ICD-11, was adopted by the 72nd World Health Assembly in 2019 and came into effect on 1 January 2022. ICD-11 aims to improve clinical, epidemiological, and population health management processes by providing a more detailed and contemporary classification of diseases and health conditions. This transition is not only critical for developed countries but also holds substantial implications for healthcare in complex humanitarian settings and resource-constrained healthcare environments.

The United Nations Relief and Works Agency for Palestine Refugees (UNRWA) was one of the earliest healthcare systems globally to implement ICD-11 in its cloud-based electronic medical record (EMR) system across 140 primary care clinics serving 5.9 million Palestine refugees. The significance of this initiative extends beyond operational improvements; it offers insights into the facilitation of health system transformation in resource-constrained environments.

Previous studies have demonstrated the challenges of medical data classification in humanitarian settings. These challenges include the difficulty of maintaining consistent and reliable data collection amid conflicts, natural disasters, and other crises that often disrupt healthcare infrastructure. In such environments, the lack of standardization, the need for rapid response, and the precarious conditions under which health data must be collected further complicate efforts to classify and utilize medical data effectively [1,2].

Research into the adoption of ICD-11 has primarily focused on its technical and infrastructural implications [3]. However, debates persist regarding the practical challenges of implementing such sophisticated systems in resource-constrained environments, with concerns about data privacy, system complexity, physician engagement, and the sustainability of training and support [4,5,6]. These issues highlight the need for comprehensive preparations that not only address the technical and logistical aspects of ICD-11 integration but also consider the sociopolitical contexts that influence these processes.

The purpose of this article is to examine the pilot implementation of ICD-11 within UNRWA’s health infrastructure, identifying barriers and facilitators encountered during this process. This article contributes to the broader discourse on improving health information systems in humanitarian and resource-constrained settings, providing actionable learnings that can inform similar initiatives worldwide.

## 2. Methodology

This section provides an overview of the phases involved in adopting ICD-11, the specific data points collected, and the analytical tools used to evaluate the implementation.

### 2.1. Settings and Participants

This pilot study was conducted in all 140 primary care clinics operated by UNRWA. The focus was on evaluating the integration of ICD-11 into UNRWA’s existing electronic medical records (EMR) system.

The study involved medical officers (MOs) who were responsible for implementing and utilizing the new classification system, as well as the patients whose consultations were documented using ICD-11.

### 2.2. ICD-11 Implementation Phases

The rollout of ICD-11 was structured into four phases: Stakeholder Alignment, Preparation, Training, and Implementation [7]. Stakeholder alignment: This phase commenced with a three-day meeting organized by the WHO Regional Office for the Eastern Mediterranean (EMRO) in Luxor, Egypt, in January 2019. The meeting, held in collaboration with the Egyptian Ministry of Health and Population, aimed to orient health professionals from the region to ICD-11. Participants also had the opportunity to discuss anticipated challenges and reach a consensus on using dual coding (ICD-10 and ICD-11) during a designated transition period. Participants also agreed on the necessity of conducting field trials, or pilots, prior to a full rollout.

Preparation: Following the Luxor meeting, UNRWA’s Department of Health held a series of internal meetings in Amman, Jordan, to determine their own strategy for transitioning clinics from ICD-10 to ICD-11. A task force was established, comprising members from the Department of Health, as well as the Information Technology and Management Department (IMTD). The task force began by conducting a technical evaluation of the existing EMR system and developed a formal scope of work outlining the required upgrades. The scope of work also suggested initial modules to pilot ICD-11 in, including outpatient services, non-communicable disease, maternal and child health, dental services, mental health, laboratory, and pharmacy. Ultimately, outpatient services were selected for the pilot study, because of their comprehensiveness of common illnesses. In order to safeguard all personal health information during and after the transition, the IMTD developed an integrated container technology (using WHO docker containers). The IMTD also incorporated application programming interfaces (APIs), so as to maintain access to the most current ICD-11 codes on WHO servers [8].

Design and development: Once the IT infrastructure was in place, the outpatient module was developed for the pilot study. User-centered design principles were applied to these processes, in which the end users (MOs) influenced the design throughout its development. This approach helped to ensure the new module would be intuitive and clinically useful. For example, rather than displaying a main page with a navigation bar to locate ICD-11 codes, the module was designed to guide MOs through a four-step workflow that mirrored their interaction with the patient. This included a click-through of the initial assessment (vital signs), chief complaints (signs and symptoms reported by the patients), medical examinations performed, diagnosis, and prescription. Overall, six features were either added or upgraded in response to input from Mos, namely the following:

1. The feature “Chronic known conditions” was added to the patient chart, along with information about their medications, any special instructions, or potential side effects;
2. An API-enabled search bar was added for quickly locating the ICD-11 code associated with the chief complaint(s).
3. “Type of visit” was added to the patient chart, which informed MOs about their patients’ medical histories, current medical conditions, and any planned follow-up visits. This field prompts MOs to select either “New visit” or “Follow-up visit” and effectively creates a log of records. This is particularly useful when managing patients with chronic conditions, who often require multiple visits for the same condition.
4. A snapshot of patients’ previous visits was added within the main consultation screen, thus enabling MOs to see a high-level view of the visit history without needing to navigate away from the main dashboard.
5. A search bar was added for “Diagnosis or reason for encounter”. Like the search bar for locating the ICD-11 code associated with the patients’ chief complaint, this feature enables MOs to enter a description of the diagnosis and select the appropriate code.
6. Alerts were generated for pharmacists when dispensing medications to patients, including instructions for speaking with the patients about their prescriptions.

The outpatient module, along with its additional features, underwent rigorous quality assurance testing to refine the system and rectify any bugs. The testing was conducted using a test environment link, which was distributed to sites in each operational area—Jordan, Syria, Lebanon, Gaza, and the West Bank—as well as to Department of Health staff.

Training: A series of training programs were developed to build capacity among MOs. These included live workshops and e-learning modules, the latter being critical due to emerging COVID-19 protocols. The first live workshop was delivered to 50 MOs in June 2019 in partnership with the WHO. The second was delivered to an additional 70 MOs in March 2020. Due to emerging COVID-19 protocols, the live workshops could not be continued, and the training was adapted to a fully virtual format. The IMTD identified Moodle as a training platform and held simulation exercises in uPerform. The first virtual training was piloted among 78 MOs in July 2020, and their feedback was integrated into a final version of the course that was rolled out to the remaining MOs in August 2020. At the end of the training period, all of UNRWA’s 400 MOs underwent an assessment and were awarded certificates upon achieving a minimum passing score of 80%.

Implementation and monitoring: The ICD-11 integrated outpatient module was piloted in September 2020 in all 140 of UNRWA’s clinics and was supported by continuous monitoring and evaluation.

### 2.3. Data Collection and Analysis

Data quality, system usability, and user satisfaction were assessed through surveys, interviews, and detailed reports. These reports, generated in Microsoft PowerBI by the Department of Health in collaboration with the IMTD, offered insights into various aspects of population health. The reports covered a wide range of metrics, including demographic data; environmental factors influencing disease distribution; and the relationships between patients’ diagnoses, prescribed medications, and other relevant variables. To facilitate targeted analysis, the reports were disaggregated by sex, age, and multiple classifications across different clinics and operational areas, providing a granular view of the data. The surveys were designed to gain a deeper understanding of the MOs’ experience with the new system. Interviews were conducted among the Department of Health to identify perceived barriers and facilitators to adoption.

### 2.4. Limitations

While this study provides valuable insights into the implementation of ICD-11, it is important to acknowledge certain limitations. These include potential biases in self-reported data from the MOs, challenges in data collection due to the COVID-19 pandemic, and the constraints of working within a resource-limited setting. Further research is needed to explore the long-term impacts of ICD-11 integration and to address any ongoing challenges identified during the pilot phase.

## 3. Results

This section presents the results of the implementation and integration of ICD-11 within UNRWA’s primary care system. The transition from ICD-10 to ICD-11 was intended to address several limitations associated with the former classification system, including frequent data entry errors and inconsistencies in disease categorization. These issues highlighted the need for a more precise and comprehensive system. With the introduction of ICD-11, significant improvements in data quality and system efficiency were anticipated. To fully appreciate the impact of ICD-11, it is essential to first understand the specific challenges posed by ICD-10. Prior to the transition, data entry errors were common, with notable issues in spelling and the misclassification of disease categories. For instance, “urinary tract infections” were variously entered, leading to inconsistencies in the data (Table 1). These challenges underscored the limitations of ICD-10 and set the stage for the improvements observed with the adoption of ICD-11.

### 3.1. Improvements in Data Quality and System Efficiency

The transition to ICD-11 improved the classification and documentation of medical data. Under ICD-10, diagnoses were limited to chapter-level classifications (e.g., “diseases of the nervous system”, “diseases of the respiratory system”, “certain conditions arising in the perinatal period”), which constrained the depth of patient records and reduced their utility for data analysis teams and public health administrators. The system lacked essential functionalities, such as advanced search capabilities, the ability to document principal and secondary complaints and diagnoses, compatibility with outpatient services, and the ability to generate meaningful reports. These deficiencies resulted in data that were neither sufficiently detailed nor comprehensive, limiting our ability to make comparisons with the richer classification system introduced by ICD-11.

Therefore, our analysis began in 2021, as this marked the first full year of available data following the initial implementation of ICD-11, providing a baseline for assessing its impact. The transition to ICD-11 led to significant improvements. The number of chief complaints classified increased from 5,557,298 in 2021 to 6,431,930 in 2022, representing a 15% increase (Table 2). Similarly, the number of diagnoses classified rose from 5,360,234 in 2021 to 6,161,646 in 2022, marking a 14% increase (Table 3). These increases suggest growing familiarity and proficiency with the system among MOs. Additionally, patient consultations grew from 5,234,349 in 2021 to 5,622,439 in 2022, a 7% increase (Table 4). Notably, the rate of increase in classified chief complaints and diagnoses outpaced the overall growth in patient consultations, reflecting improved documentation processes and more comprehensive use of the ICD-11 system. For example, the average number of diagnoses classified per consultation increased from 1.02 in 2021 to 1.2 in 2022 and 2023, underscoring the additional degree of detail recorded during each patient consultation. In 2023, there was a decline in the number of classified chief complaints, diagnoses, and patient consultations, primarily due to significantly reduced entries from the Gaza operational area following the onset of conflict in October 2023. This reduction was driven by damage to infrastructure and the urgent need for critical aid and relief for much of the population. As a result, UNRWA clinics in the region were either forced to operate offline or close entirely, leading to approximately 45% fewer ICD-11 entries from this area. This disruption significantly impacts year-over-year comparisons and may not accurately reflect a genuine decline in system usage or healthcare activity. Instead, it underscores the challenges of maintaining consistent data collection in conflict-affected environments, where manual patient consultation reporting with a much more simplified coding structure becomes necessary. 

In response, UNRWA prioritized gathering the most essential data needed to inform immediate health responses, with less emphasis on detailed documentation of chronic diseases, for example. This situation highlights the need for flexibility in health information management during conflicts and in war zones. While ICD-11 offers significant advantages in data quality and precision, its reliance on electronic systems makes it vulnerable in settings where connectivity is compromised. In such cases, health systems must adapt by simplifying processes to ensure that critical health information is recorded, even if in less detail.

### 3.2. Female Healthcare Engagement and Classification Trends

Female healthcare engagement consistently surpassed that of males. In 2020, a total of 705,612 chief complaints were classified for females, compared to 445,330 for males, with similar trends observed in diagnoses (696,313 for females and 432,068 for males). This pattern continued into 2021 and 2022, with the gap widening in favor of females. Although the overall numbers decreased in 2023, classifications for females remained higher, with 3,470,507 chief complaints and 3,368,563 final diagnoses, compared to 2,251,060 chief complaints and 2,149,046 final diagnoses for males. Potential explanations for this include a higher frequency of visits related to child and maternal care or a broader range of health conditions among females. Additionally, the structure of health programs within UNRWA clinics, which feature specific outreach and targeted programs for women, particularly in areas such as perinatal care and child vaccination, likely contributes to these observed trends.

### 3.3. Adoption and User Experience

One-hundred and three MOs completed an online, self-administered comparative evaluation survey that was designed to assess and compare their experiences with the use of ICD-11 versus ICD-10. The survey aimed to evaluate the effectiveness, challenges, and benefits of transitioning to the new system. The survey was open between 15 September and 11 October 2022 and was distributed to MOs across all five operational fields—Jordan, Syria, Lebanon, Gaza, and the West Bank. It focused on several aspects, including the following:

1. The level of detail recorded with ICD-11 compared to ICD-10;
2. The frequency of mistakes made using ICD-11 versus ICD-10;
3. The impact of ICD-11 on the ability to make rational treatment decisions;
4. The effect of ICD-11 on clinical workflow—whether it simplifies or complicates it;
5. The time spent entering clinical recordings with ICD-11 compared to ICD-10;
6. The ease of use of ICD-11, rated on a scale;
7. Whether the respondents received formal ICD-11 training.

Overall, the findings suggest that most respondents (81.6%) found ICD-11 to allow for more detailed clinical recordings, and 58.3% noted that it resulted in fewer mistakes compared to ICD-10. However, there was mixed feedback regarding its impact on clinical workflow and the time spent entering recordings, with many reporting that ICD-11 complicated their workflow (39.8%) and required more time for data entry (65.0%). Additionally, the ease-of-use ratings were moderate, with 42.7% giving a rating of 3 out of 5, indicating room for improvement in user experience with ICD-11.

In addition to the main findings, the survey revealed other positive aspects of ICD-11. Notably, 45.6% of respondents reported that using ICD-11 improved their ability to make rational treatment decisions. This suggests that nearly half of the respondents found ICD-11 beneficial in aiding their clinical judgment. Furthermore, 15.5% of respondents indicated that they could see more patients per day when using ICD-11, suggesting that for some, the new system has improved efficiency in patient management. These positive outcomes underscore the benefits perceived by certain users in adopting ICD-11.

### 3.4. Barriers and Facilitators to Implementation

Interviews were conducted with UNRWA Health Department staff to identify the key barriers and facilitators during the implementation of ICD-11 (Table 5 and Table 6). Barriers ranged from technical issues such as data security and system interoperability to human actors like staff turnover and the capacity for epidemiological data analysis. Conversely, facilitators included various forms of support and training provided, commitment from leadership, and the enabling environment created through policy support and international recognition.

## 4. Discussion

The implementation of ICD-11 within UNRWA’s primary care system represents a pivotal advancement in health data management in complex humanitarian settings. This study reveals several key findings that underscore the transformative potential of ICD-11, particularly in improving data quality, system efficiency, and clinical documentation practices.

Firstly, the transition from ICD-10 to ICD-11 significantly enhanced the precision of health data classification. The limitations of ICD-10, which often resulted in data entry errors and inconsistencies, were effectively addressed by ICD-11’s more detailed classification system. The 15% increase in classified chief complaints and the 14% increase in diagnoses from 2021 to 2022 indicate growing proficiency among medical officers (MOs) and demonstrate the system’s capacity to capture a broader range of clinical data. These improvements are particularly important for public health surveillance and resource allocation in refugee settings, where accurate data are critical for effective health interventions.

Moreover, the analysis highlights a consistent gender disparity in healthcare engagement, with female patients consistently exhibiting higher rates of healthcare utilization. This finding points to the importance of tailored healthcare programs that address the specific needs of women in these settings, particularly in areas such as maternal and child health. Understanding these gender dynamics is crucial for designing more inclusive and effective healthcare interventions.

Despite these successes, the implementation of ICD-11 was not without challenges. The additional time required for data entry, as reported by 65% of MOs, suggests that workflow efficiency may be impacted, potentially leading to clinician fatigue or reduced time for patient interaction. Additionally, the mixed feedback on the system’s ease of use indicates that further refinements are necessary to improve user experience and ensure sustained adoption of the system.

The identification of barriers, such as data security concerns and the complexity of the classification schema, underscores the need for robust technical infrastructure, ongoing training, and adaptation to humanitarian contexts. Conversely, the facilitators identified—such as strong leadership commitment, effective communication, and continued on-site support—played a critical role in navigating these challenges and ensuring the successful integration of ICD-11.

## 5. Conclusions

In conclusion, the pilot implementation of ICD-11 within UNRWA’s primary care system represents a significant leap forward in advancing health data management in complex humanitarian settings. This transition has resulted in marked improvements in data quality, system efficiency, and user satisfaction—key factors driving healthcare delivery, particularly in resource-constrained environments. These improvements not only reflect the capabilities of ICD-11 but also underscore the importance of accurate, comprehensive clinical documentation in supporting public health initiatives and data-driven resource allocation.

However, the challenges identified during the implementation—especially those related to workflow efficiency and system usability—emphasize the need for ongoing support and iterative refinements. Moving forward, the lessons learned from this study can guide future implementations of ICD-11 and similar advanced classification systems, such as the International Classification of Health Interventions (ICHI) and the International Classification of Functioning, Disability, and Health (ICF), in other humanitarian contexts [10].

Moreover, integrating ICD-11 with pharmacy information offers significant potential for improving patient care. By linking medication data with specific diagnoses, healthcare providers can gain a more comprehensive understanding of treatment patterns and outcomes. This integration is particularly valuable in resource-limited settings, where it can facilitate more targeted and effective interventions.

By addressing the barriers and capitalizing on the facilitators identified in this study, policymakers and healthcare practitioners can fully harness the potential of ICD-11 to enhance health outcomes and promote health equity for vulnerable populations globally. Further research is essential to explore the long-term impacts of ICD-11 integration and to assess its scalability in other low-resource settings, ensuring that these advancements continue to support the overarching goal of health equity worldwide.

## Figures and Tables

**Table 1:** Data inconsistencies in urinary tract infections classification in ICD-10.

Chief Complaint or Diagnosis	Inconsistency
UTI?	Unnecessary use of question mark
Recurrent UTI	Use of “Recurrent”, synonymous with “Chronic”
Sever UTI	Misspelling of “Severe”
FU of UTI	Non-standardized acronym for “follow-up”
Chronic UTI	Use of “Chronic”, synonymous with “Recurrent”
?UTI	Unnecessary use of question mark
Uti??	Unnecessary use of question mark

**Table 2:** Number of chief complaints documented with ICD-11.

Period	Chief Complaints Classified		
	Male	Female	Total
Sep-Dec 2020	445,330	705,612	1,150,942
Jan-Dec 2021	2,215,089	3,342,209	5,557,298
Jan-Dec 2022	2,558,141	3,873,789	6,431,930
Jan-Dec 2023	2,251,060	3,470,507	5,721,567

**Table 3:** Number of diagnoses classified with ICD-11.

Period	Diagnoses Classified		
	Male	Female	Total
Sep-Dec 2020	432,068	693,313	1,125,381
Jan-Dec 2021	2,113,583	3,246,651	5,360,234
Jan-Dec 2022	2,422,091	3,739,555	6,161,646
Jan-Dec 2023	2,149,046	3,368,563	5,517,609

**Table 4:** Number of patient consultations entered with ICD-11.

Period	Patient Consultations (Total)
Sep-Dec 2020	1,029,953
Jan-Dec 2021	5,234,349
Jan-Dec 2022	5,622,439
Jan-Dec 2023	4,666,416

**Table 5:** Barriers to implementation identified by the UNRWA Department of Health.

Barriers	
Data security and interoperability issues	IT infrastructure required certain measures to safeguard sensitive information, such as utilizing Docker container technology to enable offline use of the ICD-11 system. This is run on WHO APIs that are updated periodically.
Complex classification schema	Classification schema beyond the scope of practice for UNRWA clinicians in humanitarian settings.
Frequent staff turnover	Due to recent budget cuts, UNRWA had to rely on short-term contract employees, particularly within the IMTD, which was a barrier to the continuity and sustainability of knowledge and projects.
Limited capacity to analyze data for epidemiological purposes	Adoption of new methods capable of handling a greater level of detail, including disease distribution, risk factors, and associations between patients, diagnoses, medications, and other personal health information.

**Table 6:** Facilitators to implementation identified by the UNRWA Health Department.

Facilitators	
Sufficient training	Providing sufficient training to providers to ensure that they understand the new coding system and how to use it effectively, including training in both English and Arabic using hybrid learning technologies [9].
Continued on-site support	Continued technical support from the ICD-11 taskforce, IMTD, and EMRO helped to ensure the successful adoption and sustained use of ICD-11.
Interoperability measures	UNRWA put in place the needed infrastructure to support ICD-11 integration into the HER system, leveraging existing WHO-developed APIs.
Clear and effective communication about the new system	Providing clear and effective communication to providers about the new coding system, including mechanisms to collect real-time feedback and work with providers to overcome barriers.
Degree of willingness and acceptance among clinicians	Due to UNRWA’s long-standing collaboration with the WHO and position as a recognized partner, MOs are highly familiar with and accepting of WHO training and tools and can adapt quickly to implement new systems and technologies.
Leadership commitment and effective change management	UNRWA has a systematic approach to transitioning priorities, processes, and technologies. Leadership support and an organizational culture familiar with iterative improvement cycles were additional facilitators.
Policy support and recognition from international organizations	Policy support and recognition from international organizations, including the WHO and EMRO, to promote the adoption and use of ICD-11.

## Data Availability

The datasets presented in this article are not readily available due to privacy and ethical restrictions. Requests to access the datasets should be directed to Wafa’a Zeidan at the UNRWA Health Department. Such requests must include appropriate justification and will be reviewed by the UNRWA Research Review Board in accordance with UNRWA policy.
